# Dasatinib rapidly induces deep molecular response in chronic-phase chronic myeloid leukemia patients who achieved major molecular response with detectable levels of *BCR*-*ABL1* transcripts by imatinib therapy

**DOI:** 10.1007/s10147-017-1141-y

**Published:** 2017-05-26

**Authors:** Masayuki Shiseki, Chikashi Yoshida, Naoki Takezako, Akira Ohwada, Takashi Kumagai, Kaichi Nishiwaki, Akira Horikoshi, Tetsuya Fukuda, Hina Takano, Yasuji Kouzai, Junji Tanaka, Satoshi Morita, Junichi Sakamoto, Hisashi Sakamaki, Koiti Inokuchi

**Affiliations:** 1 Department of Hematology, Tokyo Women’s Medical University, 8-1 Kawadacho, Shinjuku-ku, Tokyo, 162-8666 Japan; 2 Department of Hematology, National Hospital Organization Mito Medical Center, Ibaraki, Japan; 3 Department of Hematology, National Disaster Medical Center, Tokyo, Japan; 4 Department of Hematology, Tokyo Metropolitan Bokutoh Hospital, Tokyo, Japan; 5 Department of Hematology, Ohme Municipal General Hospital, Tokyo, Japan; 6 Clinical Oncology and Hematology, Jikei University Kashiwa Hospital, Chiba, Japan; 7 Department of General Internal Medicine, Nerima-Hikarigaoka Hospital, Tokyo, Japan; 8 Department of Hematology, Tokyo Medical and Dental University, Tokyo, Japan; 9 Department of Hematology, Japanese Red Cross Musashino Hospital, Tokyo, Japan; 10 Hematology Department, Tokyo Metropolitan Tama Medical Center, Tokyo, Japan; 11 Department of Biomedical Statistics and Bioinformatics, Kyoto University Graduate School of Medicine, Kyoto, Japan; 12 Tokai Central Hospital, Gifu, Japan; 13 Hematology Division, Tokyo Metropolitan Cancer and Infectious Diseases Center, Komagome Hospital, Tokyo, Japan; 14 Division of Hematology, Department of Internal Medicine, Nippon Medical School, Tokyo, Japan

**Keywords:** Chronic myeloid leukemia, Deep molecular response, Dasatinib

## Abstract

**Background:**

With the introduction of imatinib, a first-generation tyrosine kinase inhibitor (TKI) to inhibit BCR-ABL1 kinase, the outcome of chronic-phase chronic myeloid leukemia (CP-CML) has improved dramatically. However, only a small proportion of CP-CML patients subsequently achieve a deep molecular response (DMR) with imatinib. Dasatinib, a second-generation TKI, is more potent than imatinib in the inhibition of BCR-ABL1 tyrosine kinase in vitro and more effective in CP-CML patients who do not achieve an optimal response with imatinib treatment.

**Methods:**

In the present study, we attempted to investigate whether switching the treatment from imatinib to dasatinib can induce DMR in 16 CP-CML patients treated with imatinib for at least two years who achieved a major molecular response (MMR) with detectable levels of *BCR*-*ABL1* transcripts.

**Results:**

The rates of achievement of DMR at 1, 3, 6 and 12 months after switching to dasatinib treatment in the 16 patients were 44% (7/16), 56% (9/16), 63% (10/16) and 75% (12/16), respectively. The cumulative rate of achieving DMR at 12 months from initiation of dasatinib therapy was 93.8% (15/16). The proportion of natural killer cells and cytotoxic T cells in peripheral lymphocytes increased after switching to dasatinib. In contrast, the proportion of regulatory T cells decreased during treatment. The safety profile of dasatinib was consistent with previous studies.

**Conclusion:**

Switching to dasatinib would be a therapeutic option for CP-CML patients who achieved MMR but not DMR by imatinib, especially for patients who wish to discontinue TKI therapy.

## Introduction

Chronic myeloid leukemia (CML), a neoplastic disorder of hematopoietic stem cells, is caused by a BCR-ABL1 fusion protein that results from t(9;22)(q43;q11). With the introduction of imatinib, a first-generation tyrosine kinase inhibitor (TKI), to inhibit BCR-ABL1 kinase, the outcome of chronic-phase CML (CP-CML) has improved dramatically [1, 2]. However, only up to 10–15% of CP-CML patients could achieve a deep molecular response (DMR) after 2 years of imatinib treatment [3, 4]. Although definitions of DMR are varied among clinical trials, it is considered to be *BCR*-*ABL1* transcript levels <0.01% (MR4) on the International Scale (IS), as measured by the standardized quantitative real-time polymerase chain reaction (RQ-PCR) method [5].

Treatment with second-generation TKIs, dasatinib and nilotinib, has shown faster and deeper responses in newly diagnosed CP-CML patients than imatinib [6, 7]. Dasatinib is 325 times more potent than imatinib in the inhibition of BCR-ABL1 tyrosine kinase in vitro [8]. In addition, dasatinib shows unique immunological activity which is not observed in other TKIs. An increased number of large granular lymphocytes (LGLs) is observed in a substantial proportion of CML patients treated with dasatinib [9–12] and is associated with a superior clinical response [9–11, 13–15]. These facts indicate that dasatinib has immunological anti-leukemic effects in addition to the direct action on leukemic cells thorough BCR-ABL1 tyrosine kinase inhibition.

Dasatinib is effective and induces a deeper response in CP-CML patients who do not achieve an optimal response with imatinib treatment. We hypothesized that dasatinib can induce DMR in CP-CML patients who achieve a major molecular response (MMR) corresponding to 0.1% IS (MR3), but not DMR with imatinib treatment, through more potent TKI activity and unique immunological properties related to anti-leukemic effects. Therefore, we conducted a clinical study in which dasatinib replaced imatinib in CP-CML patients who achieved MMR but not DMR after at least two years of imatinib treatment.

## Patients and methods

### Patients

Inclusion criteria for the study were patients with CP-CML who were aged ≥20 years, treated with imatinib for at least 24 months and achieved MMR with detectable levels of *BCR*-*ABL1* transcripts by RQ-PCR, and a sensitivity of at least MR4 below the standardized line. Exclusion criteria were patients with a performance status (PS) of grade ≥3 by ECOG definition, with active second primary cancer, with clinically defined pleural effusion, with a history of critical cardiovascular events, including acute myocardial infarction within 6 months, angina pectoris within 3 months, and congestive heart failure within 3 months, with prolonged QTc of ≥450 ms or the possibility of congenital prolonged QT syndrome, with a history of treatment by dasatinib, or with *ABL1* mutations (T315I, F317L and V299L) for which dasatinib is ineffective, at the time of study inclusion. Pregnant or breastfeeding women were also ineligible.

### RQ-PCR analysis for BCR-ABL1 transcripts

The *BCR*-*ABL1* transcript level in peripheral blood was monitored before and at 1, 3, 6, 9, 12, 15 and 18 months after switching to dasatinib. To measure the *BCR*-*ABL1* transcript level, the RQ-PCR method with at least MR4 sensitivity (i.e., 0.0069% on IS) was carried out by Bio Medical Laboratories (BML, Inc., Tokyo, Japan) as described previously [14, 16, 17]. In this analysis, we defined DMR as a peripheral major *BCR*-*ABL1/ABL1* transcript ratio below the detection limit of RQ-PCR analysis.

### Screening for BCR-ABL1 mutations

Before switching to dasatinib, we screened for 25 clinically important *BCR*-*ABL1* mutations at 18 nucleotide positions by RT-PCR using PCR-Invador assay (BML, Inc., Tokyo, Japan) in all enrolled patients [18, 19].

### Study design and treatment

After confirmation of MMR with detectable levels of *BCR*-*ABL1* transcripts by RQ-PCR, dasatinib therapy was initiated for eligible patients at a dose of 100 mg once a day. The study treatment was continued until disease progression or development of unacceptable adverse events. During the study, any treatment for CML other than dasatinib was not allowed. Therapy for comorbidities and/or adverse events was permitted. Interruption, dose reduction, or dose re-escalation of dasatinib due to adverse events were allowed.

### Evaluation of efficacy

The primary endpoint in this study was the achievement of DMR at 12 months after switching to dasatinib. Secondary endpoints included the dose intensity of dasatinib at 12 months, progression-free survival, safety profiles, and immunophenotypic alterations of peripheral lymphocytes after switching to dasatinib. Progressive disease was defined as loss of complete hematological response, loss of MMR, progression to accelerated/blastic phases, or death from any cause during the treatment period.

### Evaluation of safety

Adverse events were monitored and assessed according to the Common Terminology Criteria for Adverse Events version 4.0 in all participating patients throughout the study. Chest X-ray was carried out to check pleural effusion at baseline, at 2 weeks and at 1, 2, 3, 6, 9, 12, 15 and 18 months after switching to dasatinib or more frequently if necessary. An ECG was carried out to screen for arrhythmia and to monitor the QTc interval at baseline, at 2 weeks and at 1, 2, 3, 6, 9, 12, 15 and 18 months after switching to dasatinib or more frequently if necessary.

### Monitoring immunophenotypes in peripheral blood lymphocytes during dasatinib treatment

Immunophenotypes of peripheral blood lymphocytes were monitored before, at 2 weeks and at 1, 3, 6, 9 and 12 months after switching to dasatinib by two- or three-color flow cytometry performed by BML Inc. using monoclonal antibodies against the following antigens—CD3, CD4, CD8, CD16, CD56, CD57, CD25 and CD127.

### Statistical analyses

Differences in immunophenotypes in peripheral lymphocytes between paired samples before and at 6 months after switching to dasatinib were evaluated statistically by paired *t* test using JMP Pro 11.2 (SAS Institute Inc.). A statistically significant result was considered to have a *P* value of <0.05.

### Ethics and study management

The study was conducted by the Kanto CML study group, which consists of 13 institutions in Japan, according to the Declaration of Helsinki, and the protocol was reviewed by institutional review boards or ethics committees for each participating center. Before entry into the study, all candidates were informed about the aim of the present study, and also merits and demerits of switching to dasatinib. Merits include induction of deeper response and leading to future TKI discontinuation. Demerits include newly developed adverse events caused by dasatinib, although certain adverse events due to imatinib treatment may be solved. Written informed consent was obtained from all participating patients before registration. This trial was registered at www.clinicaltrials.gov as # NCT01342679.

## Results

### Patients and treatment

A total of 19 patients were registered for the study from April 2011 to March 2013. Three patients were excluded from the study before switching to dasatinib treatment owing to withdrawal of consent. Subsequently, treatment was switched from imatinib to dasatinib in the remaining 16 patients (11 males and 5 females; median age 50 years, range 25−72 years). The characteristics of the 16 patients are summarized in Table 1. No *BCR*-*ABL1* mutations were detected at 18 nucleotide positions in all 16 participating patients before switching to dasatinib. The median time from CML diagnosis to dasatinib treatment was 56 months (range 26−173 months). All patients were PS 0 when the dasatinib treatment started. For the current analysis, all patients had follow-up periods of at least 12 months after switching to dasatinib, and the median follow-up period was 27.6 months (range 12.9–38.5 months).

**Table 1:** Characteristics of the 16 patients in the study

Age (years)	
Median	50
Range	25–72
Sex	
Male/female	11/5
Time since diagnosis of CML (months)	
Median	56
Range	26–173
ECOG performance status, *n* (%)	
0	16 (100)
1	0 (0)
2	0 (0)
Base-line *BCR*-*ABL1* transcript level (IS%)	
Median	0.014
Range	0.0054–0.078

The median and average daily doses of dasatinib at 12 months after switching to dasatinib were 99.5 mg per day and 88.0 mg per day, respectively (range 25.6–100 mg per day), and eight patients received 100% doses of dasatinib. The median treatment duration was 550 days (range 120− 691 days). Two patients (13%) discontinued the treatment within 12 months because of adverse events and patient request.

### Efficacy

**Fig. 1:**
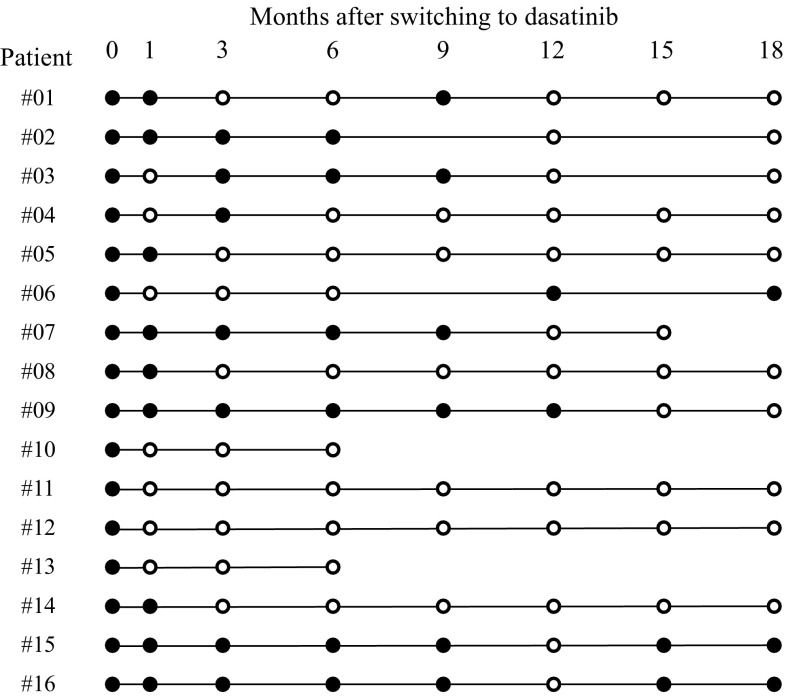
Undetectable level of *BCR*-*ABL1* transcripts during dasatinib therapy. Results of monitoring levels of *BCR*-*ABL1* fusion transcripts measured by quantitative RT-PCR with sensitivity of at least MR4.0 in 16 patients. Closed circles indicate detectable *BCR*-*ABL1* transcripts. Open circles indicate undetectable *BCR*-*ABL1* transcripts. Patients no. 10 and no. 13 discontinued dasatinib therapy within 12 months after switching to dasatinib

**Fig. 2:**
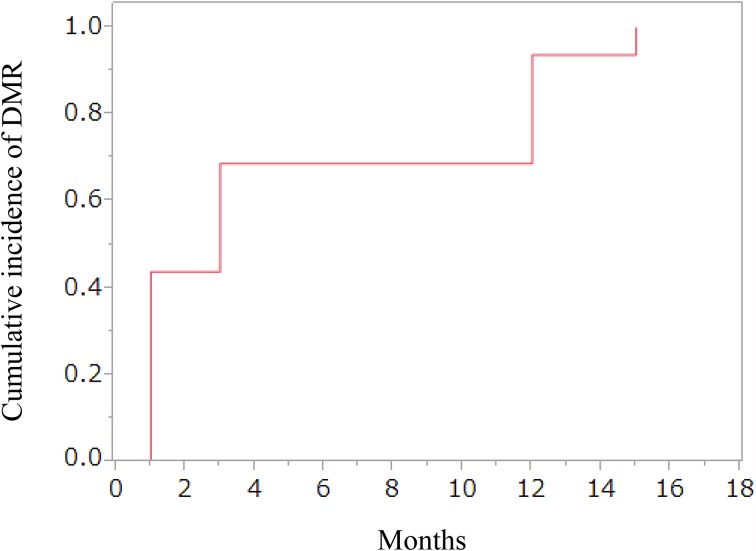
Cumulative rate of achievement of DMR by dasatinib treatment

The rates of achievement of DMR at 1, 3, 6 and 12 months after switching to dasatinib treatment in 16 patients were 44% (7/16), 56% (9/16), 63% (10/16) and 75% (12/16), respectively (Fig. 1). The cumulative rate of achieving DMR at 12 months was 93.8% (15/16) (Fig. 2). In six patients who achieved DMR, detectable levels of *BCR*-*ABL1* transcripts were observed to have re-emerged during dasatinib treatment (Fig. 1). Among these six patients, three patients subsequently obtained DMR again by continuing dasatinib treatment. At 18 months after switching to dasatinib, 10 patients (55.6%) maintained DMR. The two patients (no. 10 and no. 13) who discontinued dasatinib within 12 months achieved DMR at 6 months, although RQ-PCR data at 12 months were not available. One of these two patients (patient no. 10) was switched to nilotinib treatment and maintained DMR at 35.5 months after the initiation of dasatinib treatment. No death or disease progression was observed during the study period, and progression-free survival at 12 months was 100%.

### Safety profile

Therapy-related adverse events are summarized in Table 2. Hematologic toxicities were common adverse events, although grade 3 or 4 toxicities were less frequent. Anemia, neutropenia and thrombocytopenia were observed in 13 (81.3%), 8 (50%) and 8 (50%) patients, respectively. The most common non-hematologic adverse event was grade 1 or 2 pleural effusion, which occurred in 6 patients. The median time of appearance of pleural effusion after switching to dasatinib treatment was 9 months (range 0.5–12 months). The second most common non-hematologic adverse events were fever and skin rash, each of which occurred in 3 patients. Although grade 3 or 4 non-hematologic toxicities were rare, one male patient (patient no. 10) suffered from septicemia. He fully recovered, but subsequently discontinued dasatinib therapy. Dose reduction and/or treatment interruption due to adverse events was required in eight patients.

**Table 2:** Therapy-related toxicities

Event	All grades		Grade 3 or 4	
	No. of patients	%	No. of patients	%
Hematologic toxicities				
Neutropenia	8	50	1	6.3
Thrombocytopenia	8	50	1	6.3
Anemia	13	81.3	3	18.8
Non-hematologic toxicities				
Pleural effusion	6	37.5	0	0
Fever	3	18.8	0	0
Rash	3	18.8	0	0
Diarrhea	2	12.5	0	0
Gastro-intestinal bleeding	2	12.5	0	0
Edema	2	12.5	0	0
Septicemia	1	6.3	1	6.3
Biochemical				
Elevated AST	7	43.8	0	0
Elevated ALT	5	31.3	0	0
Elevated BUN	3	18.8	0	0
Decreased albumin	3	18.8	0	0
Hyperkalemia	2	12.5	0	0
Hypokalemia	2	12.5	0	0

Any grade 3 or 4 toxicities and/or toxicities of all grades that occurred in at least 10% of the treated patients are listed in this table

### Immunophenotypic changes in peripheral blood lymphocytes during dasatinib therapy

**Fig. 3:**
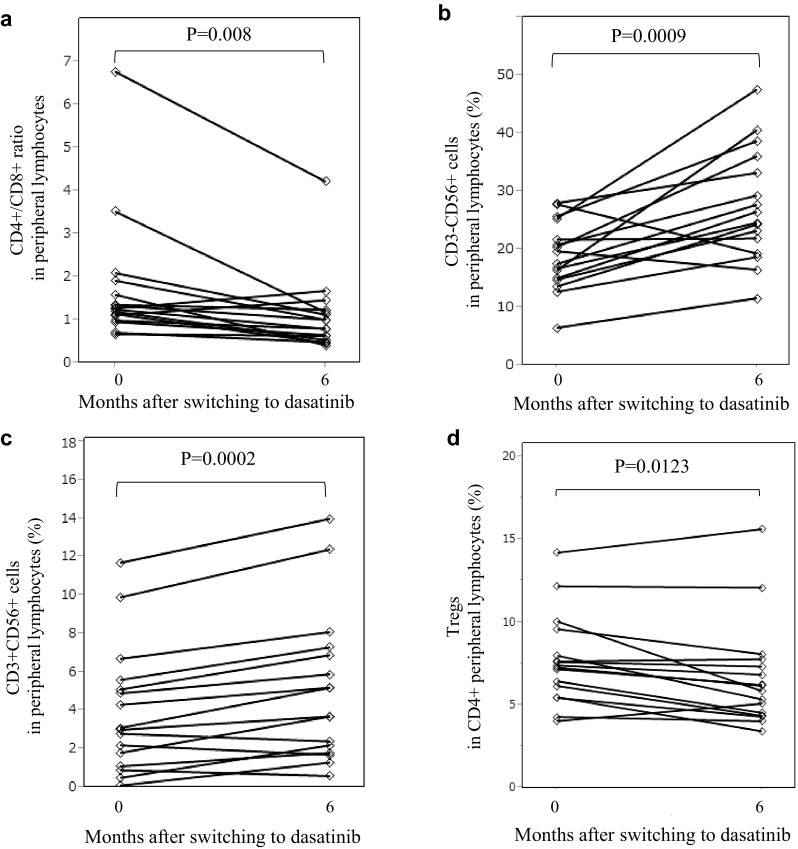
Immunophenotype alterations in peripheral blood lymphocytes before and after 6 months of dasatinib treatment. **(a)** CD4/8 ratio, **(b)** percentages of NK (CD3−/CD56+) cells and **(c)** NK (CD3+/CD56+) T cells, **(d)** percentage of Treg in CD4 lymphocytes

Immunophenotypes of peripheral blood lymphocytes were analyzed by flow cytometry in 16 patients. The median CD4/CD8 ratios before and at 6 months after switching to dasatinib treatment were 1.74 and 1.11, respectively. The CD4/CD8 ratio decreased from baseline to 6 months after the switch with a statistically significant difference (*P* = 0.008) (Fig. 3a). The median percentages of natural killer cells, defined as CD3-CD56+ cells, in peripheral blood lymphocytes before and at 6 months after switching to dasatinib were 19.0 and 27.5%, respectively (*P* = 0.0009) (Fig. 3b). The proportion of NK T cells, defined as CD3+CD56+ lymphocytes, was also elevated during dasatinib treatment. A significant difference between the median percentages of NKT cells before and at 6 months after switching to dasatinib was also observed (median percentages 4.0 vs 5.2%, *P* = 0.0002) (Fig. 3c). The proportion of regulatory T cells (Tregs), defined as CD25+CD127+ cells, in CD4+ lymphocytes was analyzed by three-color flow cytometry before and at 6 months after treatment initiation. The median percentages of Tregs in CD4+ lymphocytes before and at 6 months after switching to dasatinib were 7.7 and 6.7%, respectively, with a statistically significant difference (*P* = 0.0123) (Fig. 3d).

## Discussion

Dasatinib induces a notable response in imatinib-intolerant and -resistant CP-CML patients and also a faster and deeper response in newly diagnosed CP-CML patients [4, 7, 20]. The present study further demonstrated that dasatinib rapidly induced DMR in most CP-CML patients who had received at least two years of imatinib treatment and obtained MMR but not DMR. Switching to nilotinib, a second-generation TKI, after long-term imatinib treatment has been previously reported, showing that nilotinib enabled more patients with CML-CP to gain a DMR compared to remaining on imatinib [21]. To our knowledge, this is the first report of switching to dasatinib from imatinib with the aim of achieving DMR.

There are several possible explanations for rapid DMR induction by dasatinib in CP-CML patients who achieved MMR, but not DMR by imatinib treatment. First, the strong TKI activity of dasatinib, which is 325 times more potent against BCR-ABL1 tyrosine kinase than imatinib in vitro, may contribute to decreasing the number of residual leukemic cells in patients treated with imatinib. In addition, dasatinib has different pharmacokinetics from imatinib. Decreased expression of human organic cationic transporter 1 (hOCT1) causes reduced influx of imatinib into leukemia cells, leading to decreased intracellular concentrations of imatinib and inferior clinical outcome in CP-CML patients treated with imatinib [22, 23]. In contrast, dasatinib is not affected by hOCT1 [24]. Although we did not analyze serum imatinib concentrations and hOCT1 expression in the present study, the different pharmacokinetics and pharmacodynamics between dasatinib and imatinib may be one of the reasons for the induction of DMR in the patients.

In the molecular pathogenesis of CML, molecules other than BCR-ABL1 tyrosine kinase play certain roles. For example, dysregulation of SRC family kinases, mTOR, and p53 in CML cells has been reported, and these molecular pathways also mediate imatinib resistance [25–27]. Dasatinib inhibits multiple tyrosine kinases, including SRC family kinases, while imatinib does not [28]. Through inhibitory effects on CML cells as a multi-kinase inhibitor, dasatinib may induce a deeper response.

Dasatinib has unique properties related to immunological anti-leukemic effects. Lymphocytosis, especially an increased proportion of LGLs, is often observed in peripheral blood during dasatinib therapy and is associated with a favorable response in CP-CML patients treated with dasatinib [9–11]. This phenomenon is rarely observed during treatment with other TKIs. The immunophenotypes of the increased LGLs are either CD3−CD56+ natural killer cells or CD3+CD8+ cytotoxic T cells. A possible hypothesis for the association between a favorable response and the increased proportion of LGLs is an immunological effect on leukemic cells mediated by natural killer and/or cytotoxic T cells induced by dasatinib. Indeed, a significant difference in the immunophenotypes of peripheral lymphocytes was observed before and after switching to dasatinib in this study, which may contribute to the achievement of DMR in patients who could not obtained it by imatinib treatment. Previously, it was shown that the proportion of Tregs, which are negative regulators of the immune response in peripheral blood, was decreased in dasatinib-treated CP-CML patients. In particular, this phenomenon was observed in patients with large granular lymphocytosis [29]. In the present study, the proportion of Tregs was decreased after switching to dasatinib. Although the mechanism has not been elucidated, the decreased proportion of Tregs may result in enhancing the anti-leukemic immune response.

Toxicities from dasatinib therapy were generally not serious and were manageable. Hematologic toxicities in the present study were mostly comparable with previous studies, in which newly diagnosed CP-CML patients received dasatinib as a first-line therapy with median follow-up periods of 12 months [7, 30]. Interruption and/or dose reduction of dasatinib due to adverse events was required in half of the patients, which was comparable with previous reports [31, 32]. At 12 months, 14 of the 16 patients continued dasatinib treatment, indicating that switching from imatinib to dasatinib was tolerable in most patients. Grade 1or 2 pleural effusion occurred in six patients during the follow-up period and was manageable in all cases. Five of the six patients with pleural effusion achieved DMR at 12 months and subsequently maintained the DMR at 18 months, demonstrating that the appearance of mild pleural effusion could be associated with better clinical outcomes in dasatinib-treated patients, as reported previously [13].

In clinical practice, MMR is defined as a treatment target of CP-CML according to the guidelines [33, 34]. It has not been fully elucidated whether induction of DMR leads to the improvement of longer clinical outcomes in CP-CML patients. Several studies demonstrated that CP-CML patients who obtained DMR by TKIs were less likely to lose MMR and showed better clinical outcomes, although the definition of DMR was different among the studies [35–37]. In the German CML-Study IV, MR4.5 at 4 years was associated with better overall survival than MR3 to MR2 (IS 1.0%). However, there were no statistical differences in survival probabilities between patients achieving MR4.5 and those achieving MR4 to MR3, which corresponds to MMR. In a study conducted by a French group, significant differences were reported in long-term clinical outcomes between the patients who obtained DMR and those with MMR but not DMR during the study [36]. More importantly, achieving DMR is a necessary requirement for discontinuation of TKI therapy, which is currently of great interest in CP-CML treatment. In clinical practice, lifelong TKI treatment is recommended for CP-CML patients who obtained an optimal response, unless severe adverse events occur [33, 34]. However, long-term continuous TKI treatment implies several issues in the patients. Low-grade non-serious toxicities, including edema, GI symptoms, and muscular clumps, which affect the quality of life of the patients, are not negligible [38]. Young female patients treated with TKI should give up being pregnant. It has been shown that TKI treatment is associated with an increased risk of cardiovascular events [39]. In addition, lifelong TKI treatment substantially results in a growing medical financial burden. A French group conducted a clinical trial for imatinib discontinuation in CP-CML patients who achieved DMR by imatinib and maintained it for at least two years [40]. The results indicated that approximately 40% of patients were in treatment-free remission without molecular relapse. Several other research groups conducted imatinib discontinuation studies, and similar results have been obtained [41–45]. Cessation of dasatinib treatment in CP-CML patients who obtained DMR has been reported by a Japanese group [17]. The achievement of DMR is essential for treatment discontinuation and treatment-free remission. However, only a small proportion of CP-CML patients substantially achieve DMR by imatinib treatment. The cumulative rate of DMR by 24 months and 36 months in CP-CML patients treated with standard doses of imatinib was up to 10 and 15%, respectively [3, 4]. The present results suggest that by switching to dasatinib, patients who achieved MMR but not DMR with imatinib treatment would become candidates for TKI discontinuation.

In conclusion, switching to dasatinib would be a therapeutic option for CP-CML patients who achieved MMR but not DMR by imatinib therapy, especially for patients who wish to discontinue TKI therapy.
